# Spontaneous Functional Recovery after Focal Damage in Neuronal Cultures

**DOI:** 10.1523/ENEURO.0254-19.2019

**Published:** 2020-01-02

**Authors:** Sara Teller, Estefanía Estévez-Priego, Clara Granell, Daniel Tornero, Jordi Andilla, Omar E. Olarte, Pablo Loza-Alvarez, Alex Arenas, Jordi Soriano

**Affiliations:** 1 Departament de Física de la Matèria Condensada, Universitat de Barcelona, Barcelona 08028, Spain; 2 Universitat de Barcelona Institute of Complex Systems (UBICS), Barcelona 08028, Spain; 3 GOTHAM Lab–Institute for Biocomputation and Physics of Complex Systems (BIFI), University of Zaragoza, Zaragoza 50018, Spain; 4 Department of Condensed Matter Physics, University of Zaragoza, Zaragoza 50009, Spain; 5 Departament de Biomedicina, Facultat de Medicina, Institut de Neurociències, Universitat de Barcelona, Barcelona 08036, Spain; 6 Centro de Investigación Biomédica en Red sobre Enfermedades Neurodegenerativas (CIBERNED); 7 ICFO–Institut de Ciències Fotòniques, The Barcelona Institute of Science and Technology, Castelldefels 08860, Spain; 8 Universidad ECCI, Bogotá 111311, Colombia; 9 Departament d’Enginyeria Informàtica i Matemàtiques, Universitat Rovira i Virgili, Tarragona 43007, Spain

**Keywords:** calcium imaging, focal damage, functional recovery, laser microsurgery, network neuroscience, neuronal cultures

## Abstract

Damage in biological neuronal networks triggers a complex functional reorganization whose mechanisms are still poorly understood. To delineate this reorganization process, here we investigate the functional alterations of *in vitro* rat cortical circuits following localized laser ablation. The analysis of the functional network configuration before and after ablation allowed us to quantify the extent of functional alterations and the characteristic spatial and temporal scales along recovery. We observed that damage precipitated a fast rerouting of information flow that restored network’s communicability in about 15 min. Functional restoration was led by the immediate neighbors around trauma but was orchestrated by the entire network. Our *in vitro* setup exposes the ability of neuronal circuits to articulate fast responses to acute damage, and may serve as a proxy to devise recovery strategies in actual brain circuits. Moreover, this biological setup can become a benchmark to empirically test network theories about the spontaneous recovery in dynamical networks.

## Significance Statement

Given the sheer size of the brain, *in vitro* models in the form of neuronal cultures have emerged as a promising tool to investigate dynamic and network alterations in detail on physical damage. Here we present a new experimental paradigm based on the combination of laser microsurgery and calcium fluorescence imaging to analyze network functional alterations after a focal lesion. We show that the network is not only able to cope with damage but that the regions around the lesion core actively participate in recovery, restoring the initial network activity levels in just 15 min. Our approach offers interesting perspectives for modeling network functional loss and recovery in a number of damage actions, from stroke to degenerative disorders.

## Introduction

The functional affectations in a neuronal circuit that arise from focal damage are complex. In the brain, the traumatic loss of neuronal tissue irreversibly disables the lesioned site and silences the connectivity pathways emerging from and converging on it (Carrera and Tononi, 2014; Fornito et al., 2015). Damage leads to broad alterations in the spatiotemporal structure of neuronal dynamics that translate into functional deficits of diverse extend and severity (Alstott et al., 2009; Corbetta et al., 2015; Fornito et al., 2015; Siegel et al., 2016). The sudden activity loss, however, triggers substantial neuroplasticity, in which activity–dependent rewiring and strengthening drives functional reorganization and recovery (Murphy and Corbett, 2009; Zhu et al., 2010; van Meer et al., 2012), ultimately restoring partially or totally the altered brain functions.

Although the biological processes involved in damage and repair are well understood (Carmichael, 2016), the network mechanisms that facilitate swift response and functional recovery constitute a fundamental paradigm still to be completely understood (Majdandzic et al., 2013). These mechanisms are central to pinpoint the extent of affectation and evaluate the capacity of the circuit to restore function. Despite progress *in vivo* through animal models (Cheng et al., 2014; Lim et al., 2014), the sheer size and intricacy of brain circuits have fostered the development of *in vitro* approaches in which network damage and subsequent recovery can be examined in detail (Richard et al., 2010; Siddique and Thakor, 2014; Holloway and Gavins, 2016). At present, however, there are no dedicated *in vitro* models that can offer a high control on the delivered damage, large-scale network monitoring and detailed functional analysis of network’s behavior.

To address these limitations, here we investigate the functional restoration of rat cortical networks *in vitro* on acute focal damage, delivered through highly focused ultrashort laser pulses that produce accurate laser ablation with micrometric resolution (Vogel et al., 2005; Thayil et al., 2008). The spontaneous activity of the cortical network is monitored through high-speed, whole-network calcium imaging (Orlandi et al., 2013; Teller et al., 2014, 2015), which allows us to quantify in detail the network functional alterations on damage and map the network interactions along recovery. We observed that damage precipitated a sudden fall of the global efficiency of the network, which gradually recovered to predamage levels in ∼15 min. Recovery was mediated by an increased spontaneous activity of the regions around the lesion core, rerouting information flow to create new functional links or to strengthen existing ones. This rich plasticity evinces the capacity of the neuronal circuit to respond to damage as a global system, and hints at the existence of whole-network homeostatic mechanisms for circuit remodeling and functional restoration. To our knowledge, the study presented here is the first *in vitro* attempt to disclose the complexity of functional restoration on acute damage, and brings new opportunities to understand resilience and recovery in brain-like circuits from a network-based perspective.

## Materials and Methods

### Ethics statement

All animal procedures were performed in accordance with the Ethical Committee for Animal Experimentation of the University of Barcelona, under order DMAH-5461.

### Experimental design

#### Clustered neuronal cultures

Cortical neurons were dissected from Sprague-Dawley rat embryos at 18–19 d of development, following procedures previously described (Teller et al., 2014, 2015). Briefly, embryonic brains were dissected, cortical neurons dissociated by repeated pipetting, neurons suspended in an appropriate culture medium, and finally plated onto 13-mm glass coverslips (Marienfield-Superior) that incorporated four perforated circular cavities in a mold of polydimethylsiloxane (PDMS). Glasses and PDMS masks were attached together and autoclaved at 105°C for firm adhesion. PDMS cavities shaped minicultures that were 3 mm in diameter, 2 mm deep, and separated from one another by 1 mm. The size of the minicultures was optimized to fit two of them in the field of view of the imaging system.

The absence of adhesive proteins in the glass substrate facilitated cell motility and aggregation, and ultimately shaped a network of dense neuronal islands (clusters) connected to one another (Segev et al., 2003; de Santos-Sierra et al., 2014; Teller et al., 2015). Neurons were seeded with a density of 2500 neurons/mm^2^, providing ∼40 clusters per culture, and were incubated in plating medium [Eagle’s MEM (Invitrogen) supplemented with 5% fetal calf serum (FCS; Invitrogen), 5% horse serum (HS; Invitrogen), 1 μl/ml B27 (Sigma), 20 μg/ml gentamicin (Sigma), 1% 100× Glutamax (Sigma), and 0.6% glucose] at 37°C, 5% CO_2_ and 95% humidity up to day *in vitro* (DIV)5. The medium was then switched to changing medium [MEM supplemented with 10% HS and 0.5% 5-fluorodeoxyuridine (FUDR)] to limit glial cell division. From DIV8 onwards, cultures were maintained in final medium (MEM supplemented with 10% HS) with a periodic fluid replacement every 3 d. The neuronal cultures contained both excitatory and inhibitory connections, which were left active in all measurements to maximize spontaneous activity.

A total of *n* = 14 cultures was used in this study. They were selected from all the pool of available cultures to comply two main conditions, namely a high spontaneous activity and a similar number of clusters. All experiments were conducted at 20°C.

#### Imaging setup

Minicultures were imaged in pairs at DIV9–DIV13, a developmental stage in which the number and position of the clusters was stable and spontaneous activity high. Neuronal activity was monitored through fluorescence calcium imaging using Fluo-4-AM as Ca^2+^ probe (Teller et al., 2014, 2015). Prior recording, cultures were incubated for 25 min in a transparent, pH-stable medium (recording solution, RS) that contained 2 μg of Fluo-4 per milliliter of solution. At the end of incubation and after washing off residual Fluo-4, the cultures were transferred to an observation chamber that contained 2 ml of RS. The chamber was sealed with a glass coverslip to prevent evaporation and left 5 min in darkness for stabilization.

The observation chamber was mounted on a multimodal microscope attached to a high-speed sCMOS camera (Hamamatsu Orca Flash 4, USB3 mode) that allowed for the simultaneous imaging of two minicultures. The multimodal microscope is a modified commercial confocal microscope (Nikon C1) that integrates a femtosecond-pulsed laser source for two-photon fluorescence microscopy (Mathew et al., 2009). This pulsed laser input was optimized for accurate multiphoton microsurgery and optical manipulation/stimulation of biological samples (Santos et al., 2013).

#### Laser microsurgery

Optical surgery in combination with fluorescence imaging was achieved by setting the microscope in three progressive configuration modes, termed top EPI, laser, and bottom transmission. Fluorescence imaging of spontaneous activity was recorded using the top EPI configuration. Here, the multimodal microscope operated as an epifluorescence microscope in an upright configuration. A mercury lamp (Nikon C-HGFI) guided by an optical fiber was coupled onto the EPI-illumination port. The green fluorescence protein (GFP) filter set for Fluo-4 Ca^2+^ imaging consisted of a dichroic mirror (FF 509 FDI) with a green filter and a blue bandpass filter (HQ470/40X). Frames were acquired with a size of 534 × 254 pixels (6.5 × 3.5 mm^2^ field of view), 16-bit grayscale, and acquisition speeds in the range of 83–100 frames/s (fps).

Neuronal clusters were targeted using the laser configuration. A Ti:sapphire laser (Mira Optima 900-F, Coherent) producing an ultrashort (150 fs) near-infrared (NIR) pulsed beam, with an average power of 400 mW in the back focal plane of the objective, was delivered onto a region of 0.7 μm^2^. In this setting, the laser light was focused using a NIR-optimized water immersion objective with 1.05 numerical aperture (25×, Olympus). A shutter was incorporated between the attenuators to control the exposure time of the laser.

Transmission bright field images of the cultures were obtained through the bottom transmission configuration. To minimize the time and changes in the custom setup, the standard bottom illumination from the EPI configuration was used together with the TRITC epifluorescence cube. The emission filter of the GFP cube of the top configuration transmitted the excitation light of the TRITC cube and produced a normal transmission image of the sample.

#### Experimental procedure

Neuronal spontaneous activity was evaluated through calcium fluorescence imaging. To select the most appropriate cultures, activity was first recorded for 5 min in the four minicultures PDMS set. The pair of minicultures that contained a comparable number of clusters and exhibited similar activity was selected, and the entire glass adjusted to fit this pair in the field of view. One of the cultures was then designated as control and the other one as target, and spontaneous neuronal activity recorded in the pair for 30 min.

The multimodal microscope was next switched to the laser configuration for microsurgery on a random cluster. With assistance of a second camera (DCC 1545M, Thor Labs) and a joystick, the laser beam was positioned on the cluster and manually guided. Damage was applied along the surface and edges of the target cluster, effectually killing all its neurons and disconnecting the cluster from the rest of the network. The microscope objective had a non-negligible chromatic aberration that produced a shift between the image obtained with the camera and the ablation IR laser. This shift was compensated by moving the focal position along the *z*-axis to the plane where the damage was induced. The duration of the entire ablation operation was ∼10 min.

Finally, the microscope was reconfigured for florescence imaging and activity in the control and target culture recorded again for additional 30 min. A bright field image of the twin minicultures was taken at the end of the experiment to obtain a detailed characterization of the neuronal clusters.

All procedures were always conducted on the pair of minicultures, one acting as control and the other as target. This ensured that all experimental manipulations, such as handling of cultures or changes in the optical configuration, were experienced by both cultures. This was particularly important in the context of the laser ablation, in which the long time of the procedure as well as temperature variations associated to laser power could alter spontaneous activity. *Post hoc* data analysis showed that the control cultures exhibited stable characteristics along the experimental pipeline, and that therefore all network changes observed in the ablated culture originated from the physical damage and not from the experimental manipulations.

#### Fluorescence signal and onset times

Fluorescence recordings were first converted into individual frames using Hokawo 2.5 software (Hamamatsu). Neuronal clusters were manually selected as regions of interest (ROIs) over the images to extract their fluorescence intensity (average grayscale level) along the recorded frames. A typical experiment contained on the order of 40 ROIs with a typical size of 40 × 40 pixels. The raw fluorescence signal of each neuron *F*(*t*) was then corrected for small drifts by detrending the signal, i.e., by fitting a straight line to the baseline and subtracting it from the data. The detrended signal was then normalized as $\text{DFF}(\%)\equiv 100\times (F-F_{0})/F_{0}$, with *F*_0_ the fluorescence level of the neuron at rest. The normalized fluorescence signal was analyzed to determine the onset times of activation, characterized by a sharp increase of the fluorescence signal of the clusters. Following previously described algorithms (Teller et al., 2014, 2015), onset times were detected as the first occurrence of the crossing between the cluster’s fluorescence signal and a threshold value set as two times the average fluorescence signal of the cluster.

#### Firing sequences

Spontaneous activity in clustered networks is characterized by the concatenated activation of two or more clusters in a short time window. These activations, termed firing sequences (Teller et al., 2015), provided the basis for the computation of the effective connectivity of the networks and its modular organization. Following previous studies (Teller et al., 2014, 2015), two or more clusters belonged to the same firing sequence when their coactivation time delay was <200 ms. For simplicity, we will also use the term “firings” to refer to these firing sequences.

### Network construction

#### Effective connectivity computation

The effective connectivity was computed either along the entire recording or along a sliding time window. The degree of coupling among pairs of clusters within a firing sequence was asserted through time delays (Teller et al., 2014). In this approach, the more frequently two clusters coactivate together, the stronger their connection weight, and with the directionality of the interaction given by the temporal order of coactivation. This approach provided a connectivity matrix $A=\{a_{ij}\}$ that was thus weighted and directed. A null model was used to evaluate the significance of the inferred effective links and to normalize the connectivity matrix. The null model consisted in a random permutation of the times of the firing events of each cluster’s time series (Teller et al., 2014, 2015). This method erased the temporal correlations among firing clusters but preserved the average network activity. A total of 500 surrogates were generated, each one procuring a connectivity matrix $A^{S}=\{a_{ij}^{S}\}$. Significant links $Z=\{z_{ij}\}$ were then set according to the *z*-score

(1) $$z_{ij}=\frac{a_{ij}-〈a_{ij}^{S}〉}{\sigma_{ij}^{S}},$$

where $〈a_{ij}^{S}〉$ is the average surrogates’ weight between clusters *i* and *j*, and $\sigma_{ij}^{S}$ the corresponding standard deviation (SD). High values of *z_ij_* reflected strong cluster-to-cluster interactions. Negative *z_ij_* values indicated links that were less connected than in a random configuration, which were disregarded and set to 0. The *z*-score implementation of Equation 1 quantified the difference, in SD units, between the cluster’s raw connectivity value and the surrogates’ average value. The *z*-score defined a fixed reference to compare different cultures and experimental conditions and did not require the selection of an arbitrary threshold for significance.

The final set of effective links’ weights $W=\{w_{ij}\}$, from which all network measures were computed, was set as

(2) $$w_{ij}=\frac{z_{ij}}{\text{max}(z_{ij})},$$

thus procuring a normalized effective connectivity matrix with values in the range [0,1]. This normalization facilitated the comparison and averaging among experiments. We verified that the procured effective connectivity matrix W using our time-delays approach was similar, in number of effective links and computed network measures, to the one obtained using other approaches such as transfer entropy (Stetter et al., 2012).

#### Network dynamic evolution

Effective connectivity matrices at different time points were constructed to monitor the time-varying behavior of the clustered cultures before and after damage. A sliding window approach (Sakoğlu et al., 2010; Kiviniemi et al., 2011; Jones et al., 2012; Allen et al., 2014) was used to compute the effective connectivity matrices. Time window of length Δt (centered at time *τ*) progressively scanned the recording without overlap. The set of firing sequences within each window was then analyzed to infer the effective connectivity matrices $W^{\tau }$. The mean firing rate of the cultures before and after damage was typically four and three firings per minute, respectively. Since a minimum number of five firing sequences was required for a reliable inference of $W^{\tau }$, the window size was set in the range 2.5<Δt<4 min in both cases. An inspection of all the experiments showed that this setting provided ∼5–12 firing sequences per window. The number of windows was therefore given by T/Δt, where *T* = 30 min is the duration of the recording, leading to 9–12 windows for the analysis of the “before” and “after” damage conditions.

### Network measures

They were computed on the time-windowed effective connectivity matrices using the Brain Connectivity Toolbox (MATLAB; Rubinov and Sporns, 2010). From here on, *N* indicates the total number of nodes in the network. The ablated node was always excluded in the analysis, both before and after damage, to prevent a bias associated to network size.

#### Nodal strength and total network strength

The nodal strength *s_i_* was defined as the sum of all input and output weights to node *i*, $s_{i}=\sum_{j}w_{ij}$. The average nodal strength $\bar{s}$ was the mean of all nodal strengths, $\bar{s}=(1/N)\sum_{i}s_{i}$. The total network strength $S^{\text{net}}$ accounted for the sum of all nodal strengths or, equivalently, the sum of all weights, $S^{\text{net}}=\sum_{i}s_{i}=\sum_{ij}w_{ij}$.

#### Density of links *D*

It was defined as the fraction of total existing weighted links to all possible N(N−1) connections in the directed network. For a network with a total strength $S^{\text{net}}$, the density of links was then $D=S^{\text{net}}/(N(N-1))=\sum_{ij}w_{ij}/(N(N-1))$.

#### Global efficiency *G*

The efficiency *E* of a network of *N* nodes was calculated as (Rubinov and Sporns, 2010)

(3) $$E=\frac{1}{N(N-1)}\sum_{N=1}^{\infty }\frac{1}{d(i,j)},$$

where *d*(*i*, *j*) denotes the minimum topological distance connecting nodes *i* and *j*. The global efficiency *G* is the relative value $G=E/E_{c}$, where *E_c_* refers to the efficiency of a clique formed by the same number of nodes. *G* provided a quantification of the communication among neuronal clusters and the integration capacity of the network.

#### Time evolution of *D* and *G*

Control and ablated cultures were measured simultaneously in each recording, and analyzed identically. The time evolution of either *D* or *G* along the recording was introduced to quantify the impact of laser ablation and the recovery of the culture. Thus, *D* and *G* were analyzed along different time windows centered at *τ*, as described above. Each condition (before damage or after damage) procured ∼10–15 data points. Since the window centers *τ* varied across experiments, the curves D(τ) and G(τ) were interpolated in 1 min time steps. Data were then averaged among the *n* = 14 experiments to provide the final *D*(*t*) and *G*(*t*) curves, with t={1,2…,T} min.

#### Integrability loss Λ

It provided the relative loss in global efficiency for the ablated culture following damage. It was computed for each culture as

(4) $$\Lambda (\%)=100\times \frac{{\tilde{G}}^{\text{ bef}}-G_{0}^{\text{ aft}}}{{\tilde{G}}^{\text{ bef}}},$$

where ${\tilde{G}}^{\text{ bef}}$ is the time-averaged global efficiency of the culture before damage (with SD ${\text{SD}}_{G}^{\text{ bef}}$), and $G_{0}^{\text{ aft}}$ is the first measured value of the global efficiency just after damage.

#### Integrability recovery rate Θ and recovery time *T_R_*

Θ characterizes the typical increase of the relative global efficiency along time during recovery. It was computed for each ablated culture as $\Theta (\%)=\Lambda (\%)/T_{R}$, where *T_R_* is the time required for the culture to attain the global efficiency before damage. *T_R_* was determined as the moment in which the global efficiency along recovery $G^{\text{ aft}}(t)$ first reached ${\tilde{G}}^{\text{ bef}}-{\text{SD}}_{G}^{\text{ bef}}$.

#### Neighborhoods of clusters around damage

Six neighborhoods of progressively distant rings from damage were defined. The clusters belonging to the first neighborhood were those located at a distance below a radius $r_{C}≃0.68$ mm from the ablated cluster. This radius was set as the average intercluster separation and was the same for all cultures. The second and further neighborhoods were formed by those clusters located at a distance *r_C_* from the previous ring and away from damage.

#### Interaction probability *P*

It accounted for the probability to observe intraneighborhood and interneighborhood effective links. Conceptually, *P* rendered the capacity of a pair of neighborhood rings *R_u_* and *R_v_* to project effective connections to one another. *P* was computed as

(5) $$P(R_{u},R_{v})=\sum_{i,j\in R_{u}\cup R_{v}}w_{ij}/M,$$

where *i* and *j* are the indexes of the clusters encompassing rings *R_u_* and *R_v_*, *w_ij_* their weight, and *M* all the possible directed links that can be formed between and within *R_u_* and *R_v_*.

#### Flow of links *F* and percentage variation of flow of links F^*^

The flow of links *F* quantified the fraction of weighted links that flowed between two neighborhood rings *R_u_* and *R_v_*. *F* was computed in two steps. In a first one, the percentage $C(R_{u},R_{v})$ of links between rings *R_u_* and *R_v_* with respect to all links in which ring *R_u_* participates was calculated as

(6) $$C(R_{u},R_{v})=\frac{\text{links between rings}\;R_{u},R_{v}}{\text{all links connecting}\;R_{u}\;\text{with any other ring}}=\frac{\sum_{\left(i,j)\in (R_{u}\cup R_{v}\right)}w_{ij}}{\sum_{v\neq u}\sum_{\left(i,j)\in (R_{u}\cup R_{v}\right)}w_{ij}}.$$

In a second step, *F* was determined as $F(R_{u},R_{v})=C(R_{u},R_{v})/P(R_{u},R_{v})$, where *P* is the interaction probability. This operation established *F* as a normalized measure that facilitated the averaging among different networks. The values procured by *F* for each ring pair were used to show the behavior of the network before damage, and were denoted $F_{\text{bef}}$. The behavior of the network just after damage and in subsequent temporal windows *τ* along recovery was portrayed through the percentage variation of flow of links F^*^, given by

(7) $$F^{*}(\tau )=100\times \frac{F(\tau )-F_{\text{bef}}}{F(\tau )+F_{\text{bef}}},$$

where F(τ) and $F_{\text{bef}}$ are, respectively, the flow of links at a given temporal window *τ* and before damage. We considered only the values of $F^{*}>0$ to emphasize the flow of new effective links. This helped highlighting those rings that increased the number of effective links with respect to the predamage condition.

### Statistical analysis

Statistical and graphical analyses were conducted with Origin 9.1 and Prism 8 software packages. One–way ANOVA was used to analyze the following: (1) the differences between global efficiency and density of links before and after damage and (2) the difference in activity levels between neuronal neighborhoods along recovery. Statistical significance was designated at *p* = 0.05 for all analyses. When appropriate, data were represented and examined via box plots.

## Results

### Focal damage on neuronal cultures

**Figure 1.**
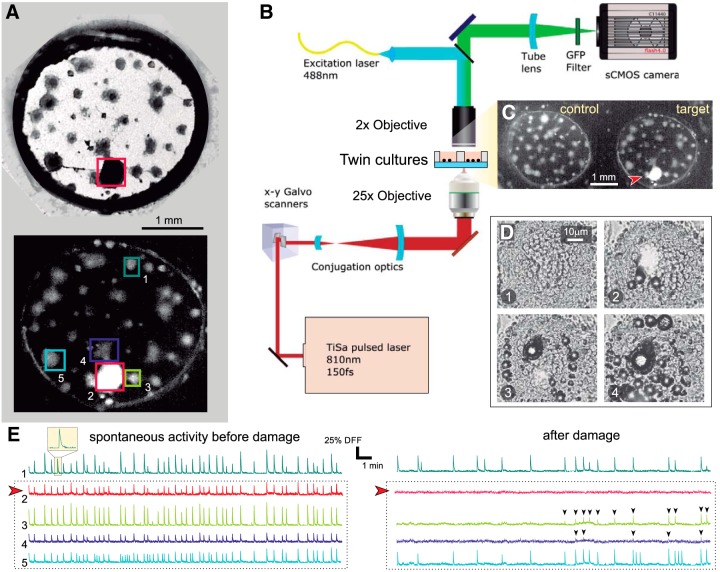
Clustered neuronal cultures and experimental procedure. ***A***, top, Bright field image of a clustered neuronal culture 3 mm in diameter. Dark circular objects are neuronal clusters, and straight filaments are connections. The ablated cluster is boxed in red. Bottom, Corresponding fluorescence image after damage. Healthy clusters appear gray. The ablated cluster, with all its neurons dead, appears bright. Boxed clusters are those whose spontaneous activity is represented in panels ***E***, ***F***. ***B***, Sketch of the multimodal optical system for fluorescence imaging and laser microsurgery. ***C***, Actual field of view in the experiments. Two cultures are simultaneously monitored, with one set as control and the other one as target. The latter is the same culture as in panel ***A***, and the red arrowhead signals the ablated cluster. ***D***, Laser microsurgery. The four snapshots illustrate the action of the laser as it progressively scans the cluster to be ablated, delivering in each step a high energy, high penetration pulse that kills the neurons and vaporizes water. The time interval between panels is 20 s. ***E***, Spontaneous activity before damage for the five clusters highlighted in ***A***. Activity is rich and all clusters fire together in a highly coordinated manner. The red arrowhead marks the cluster to be ablated. ***F***, Corresponding activity after damage, with the ablated cluster completely silent. Its immediate neighbors are initially silent but recover activity after ∼10 min, although with lower firing rates and amplitudes (black arrowheads). Clusters more distant from damage maintain their activity after ablation, although with a reduced firing rate.

We investigated the response of neuronal cultures to the destruction of a node in the network through laser ablation. Cultures were an ensemble of interconnected neuronal aggregates termed “clusters” grown on 3-mm diameter PDMS cavities. As shown in Figure 1*A*, a typical culture contained ∼40 quasi-spherical clusters with diameters in the range 50–200 μm, which connected to one another through bundles of axons that appeared as straight filaments. We monitored spontaneous activity in these cultures using fluorescence calcium imaging, a technique that revealed neuronal activations as a sharp increase in the fluorescence signal followed by a slow decay to basal levels (Fig. 1*A*,*E*,*F*).

To monitor the response of a culture to damage we used a multimodal microscope that integrated two operational modes, a first one dedicated to calcium imaging and a second one dedicated to precision laser surgery. Figure 1*B* outlines the microscope operational modes and the experimental procedure. The key advantage of the multimodal microscope is that activity monitoring and physical damage were integrated in the same system, minimizing time delays between operations and ensuring that changes in neuronal network behavior were solely ascribed to physical damage and not to other manipulations.

In a typical experiment, spontaneous activity was first monitored for 30 min in a pair of cultures (Fig. 1*C*), which were previously selected according to their similarity in number of clusters and activity. Next, one of the cultures was left unaltered as control, while the other was damaged by ablating a preset cluster from the rest of the network (Fig. 1*C*, arrowhead). Ablation was achieved through a high-power pulsed laser that scanned the entire volume of the cluster with micrometer resolution, locally increasing the temperature and generating vapor bubbles (Fig. 1*D*). Both effects led to neuronal death inside the target cluster. At the end of the process the ablated cluster appeared markedly bright and had no activity, signatures of full damage (Fig. 1*A*,*C*). The subsequent evolution of the pair of cultures was then monitored for an additional 30 min.

The changes in spontaneous activity before and after damage are shown in Figure. 1*E*,*F*, which depict the fluorescence traces for the boxed clusters of Figure 1*A*. Before damage (Fig. 1*E*), all clusters exhibited a strong coordinated activity, firing periodically together in the same time window. These episodes of high intercluster coordination reflected the strong coupling of the network. After damage (Fig. 1*F*), the trace of the ablated cluster contained just noise. Remarkably, the nearest neighbors to the ablated cluster were silent for the first 10 min to gradually restore activity afterward. The abrupt silencing of the clusters at the vicinity of the ablated one evinces the strong impact of focal damage on the immediate neighborhood, a feature that was observed in all experimental realizations. Although distant clusters decreased activity in the experiment shown here, such a long-distance affectation was rare.

### Evolution of effective connectivity after damage

**Figure 2.**
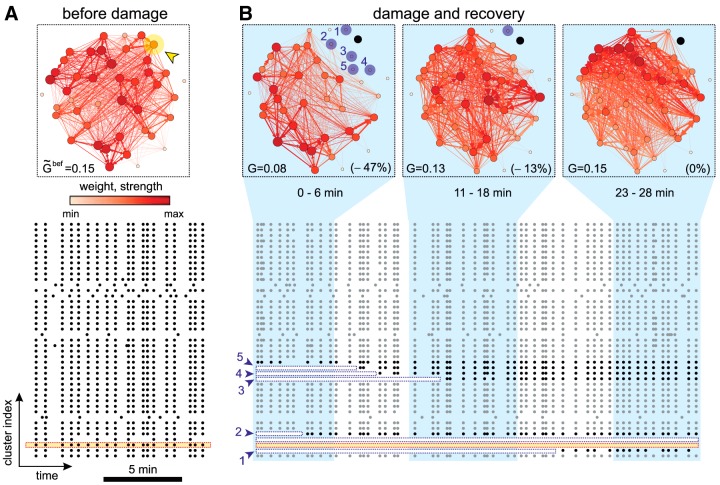
Network evolution during recovery. ***A***, Network effective connectivity and raster plot of activity before damage. The effective connectivity is computed on the full, 30 min duration of the recording. Nodes and links are color coded according to their strength and weight, respectively. The darker the color, the higher the value. The yellow arrowhead marks the targeted cluster. ${\tilde{G}}^{\text{ bef}}$ provides the global efficiency before damage. The bottom raster plot shows the 10 min before damage, with the yellow band highlighting the cluster to be ablated. Black dots are activations. ***B***, Effective connectivity evolution and raster plot after damage. The effective connectivity networks were computed in ∼6 min time windows. The ablated cluster is marked in black. Clusters in blue are those that became silent just after damage but recovered afterward, with the numbers indicating their location in the bottom raster plot. *G* provides the global efficiency, and its relative change with respect to ${\tilde{G}}^{\text{ bef}}$ is shown in brackets. In the raster plot, the ablated cluster is shown with a yellow band; the initially silent clusters are shown with a white band. One of these clusters never recovered and the band encompasses the full duration of the raster plot. Gray dots are activations in clusters that did not substantially change activity after damage. Black dots are activations in affected clusters.

To evaluate the alterations caused by damage, we considered a representative culture and computed the effective connectivity of the network along different time windows. According to the choice for effective connectivity inference, the more frequently two clusters coactivate together, the stronger the connection weight. Thus, before damage (Fig. 2*A*), the high level of coordination among firing clusters procured a strongly coupled effective network, in which the clusters with the highest strength (total incoming and outgoing weighted effective connections) were uniformly spread.

The ablation of the target cluster (Fig. 2*A*, arrowhead) precipitated different events at both spatial and temporal scales. Firstly, as shown in the raster plot of Figure 2*B*, not only the targeted cluster became silent (yellow band), but also its immediate neighbors (blue arrows, white bands). Secondly, some of these affected clusters recovered activity to levels previous to damage in ∼10–15 min, a feature that suggests the activation of fast recovery mechanisms. And thirdly, the effective networks markedly changed in organization following damage. Indeed, just after ablation the links with the highest strength appeared far from the damaged region to progressively concentrate around it. Since the strength of a node reflects its degree of interaction with neighbors, the recovery of the network is associated to an increase in intercluster activity around damage.

The behavior of the network as a whole was quantified through the global efficiency *G* (Rubinov and Sporns, 2010), which measures the degree of integrability in the network, i.e., its capacity for broad communication and information exchange. Before damage (Fig. 2*A*), the average global efficiency of the network was ${\tilde{G}}^{\text{ bef}}≃0.15$, which dropped to G≃0.08 just after damage (Fig. 2*B*). These values provided an integrability loss of ≃47%. The global efficiency gradually increased afterward and the network (excluding the ablated cluster) attained full recovery ∼15 min after damage.

### Evolution of global efficiency and density of links

**Figure 3.**
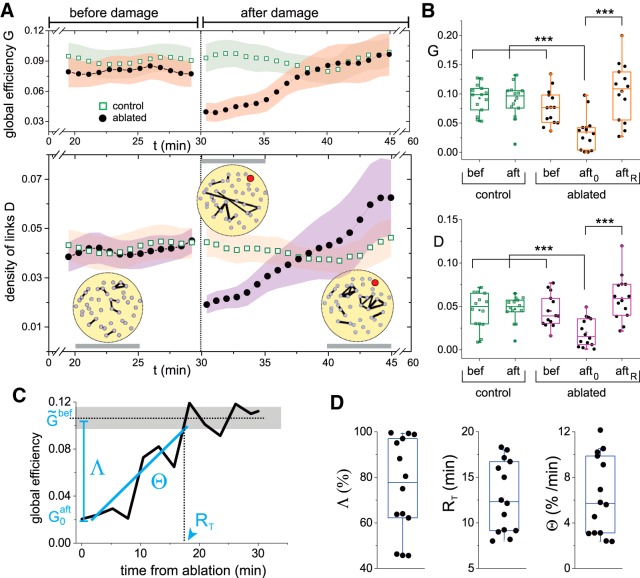
Variation of the global efficiency and the density of links on damage. ***A***, top plot, Time evolution of the global efficiency *G* for control (green) and ablated (black) cultures, before and after damage. Bottom plot, Corresponding density of links *D*. The yellow panels provide representative effective networks of the experiment shown in Figure 2, are computed over ∼5-min time windows (gray horizontal bars) and are thresholded to show the 5% links with the highest weight. The ablated cluster is marked in red. The networks illustrate the important changes in the distribution of links’ weights along the recovery process. In both plots, data were averaged over 14 cultures and the shadings show SD. For clarity, only the last 10 min before damage and the first 15 min after damage are shown. ***B***, Box plots of the distribution of *G* (top) and *D* (bottom) values for the 14 cultures at different experimental conditions, comparing controls before and after damage with ablated cultures before damage, the first 5 min after ablation (aft_0_) and the last 15 min of the recording and that correspond to the recovered state (aft*_R_*). For both *G* and *D*, significance (***p < 0.001, one-way ANOVA) is only observed between the condition just after damage and the rest of conditions. ***C***, Evolution of the global efficiency for a representative individual experiment to spotlight the definitions of the global efficiency loss Λ, recovery time *R_T_*, and recovery rate Θ. ${\tilde{G}}^{\text{ bef}}$ and $G_{0}^{\text{ aft}}$ are, respectively, the global efficiencies before damage (dotted line for average, gray shading for SD) and just after damage. ***D***, Distributions of Λ, *R_T_*, and Θ for all 14 experimental realizations. All box plots span from the median to the first and third quartiles, and whiskers span from the 10th to 90th percentile.

To prove that functional recovery was a general feature, we investigated a total of 14 cultures of identical size, containing an average number of 40 ± 5 clusters and a similar spontaneous activity of 4 ± 1 firings/min. In all cases only one node was ablated. We used the global efficiency *G* and the density of effective links *D*, averaged over experimental realizations, as main descriptors for network behavior. The global efficiency reflects whole network integrability, as seen before. The density of links, defined as the fraction of existing weighted links with respect to all possible links, portrays the degree of dynamic interactions among clusters. Figure 3*A* shows the average evolution of the global efficiency and density of links for the 14 ablated cultures and their controls. For clarity, the presented data corresponds to the 10 min before damage and to the 15 min just after damage.

The global efficiency *G* (Fig. 3*A*, top panel) exhibited a stable behavior within experimental variability, and both control and ablated cultures procured similar values of *G*. After damage, controls maintained an overall stable behavior, although fluctuations were higher probably due to the changes in the optical setup along the laser ablation procedure, which slightly increased the temperature of the recording chamber. Ablated cultures, however, experienced a substantial drop in *G* at damage, by 50%, but gradually recovered afterward and attained values of *G* very similar to predamage levels in ∼10 min. Concurrently, the density of effective links *D* (Fig. 3*A*, bottom panel) also showed a stable behavior for control and ablated cultures before damage, procuring similar values of *D* despite fluctuations. *D* dropped on damage due to the loss of activity around the ablated cluster, to gradually increase afterward as activity returned.

Both *G* and *D* exhibited strong variability on recovery (Fig. 3*A*, high SDs), with *D* even exceeding the average value before damage. We ascribe the high variability to the characteristic connectivity blueprint of each culture, which led to different interactions among clusters and therefore broad values of *G* or *D* at a given time point. On the other hand, we view the high average value of *D* as a signature of the recovery process itself, in which plasticity mechanisms that compensate for the loss of activity are activated, increasing the number and strength of effective links among clusters.

The density of links *D* exhibited interesting traits at a network level. The circular panels accompanying Figure 3*A* depict effective networks at three representative time windows of the experiment shown in Figure 2. The networks spotlight the location of the 5% strongest effective links, thus portraying the most frequent cluster’s coactivations. Before damage, the strongest links were well spread across the network and involved nearby clusters. Immediately after damage, the strongest links appeared far from the ablation core and involved distant clusters. As recovery took action, the strongest links predominated around the ablated cluster and as shortrange interactions. These changing dynamic scenarios illustrate the complexity of the recovery process and that encompasses the creation of new connectivity pathways or the strengthening of existing ones.

To show in more detail the changes in *G* and *D* in the 14 cultures before and after damage, Figure 3*B* compares in the form of box plots the behavior of the cultures at different stages. Data includes the controls before and after damage as well as the ablated cultures before damage, the window of 5 min just after damage (“aft_0_”) and the window of 15 min before the end of recording and that encompasses full recovery (“aft*_R_*”). A statistical comparison of the box plots indicated that the distributions of *G* and *D* values on ablation are significantly lower (*p* < 0.001, one-way ANOVA) than any other experimental condition. Thus, the alterations that the cultures experienced on ablation and subsequent recovery are associated to intrinsic network changes, and not to experimental details such as the number of clusters, their spatial distribution, or the culture age *in vitro*.

As an additional analysis, we quantified the degree of damage and recovery for each individual culture, and introduced three measures, namely the integrability loss Λ, the recovery time *R_T_* and the recovery rate Θ. These measures were extracted from the evolution of the global efficiency *G*(*t*) for each ablated culture, as illustrated in Figure 3*C* for one particular culture. The distributions of these measures for the 14 explored cultures (Fig. 3*D*) displayed a broad range of values. On average, the measures indicated that global efficiency decayed by ∼80% on damage, and that recovery was attained in ∼12 min at a rate of 6% increase in global efficiency per minute. We also observed that there was no correlation between any of these measures and experiment-specific characteristics such as the DIV of the culture or the location of the ablated clusters.

### Activity in the neighborhood of damage

**Figure 4.**
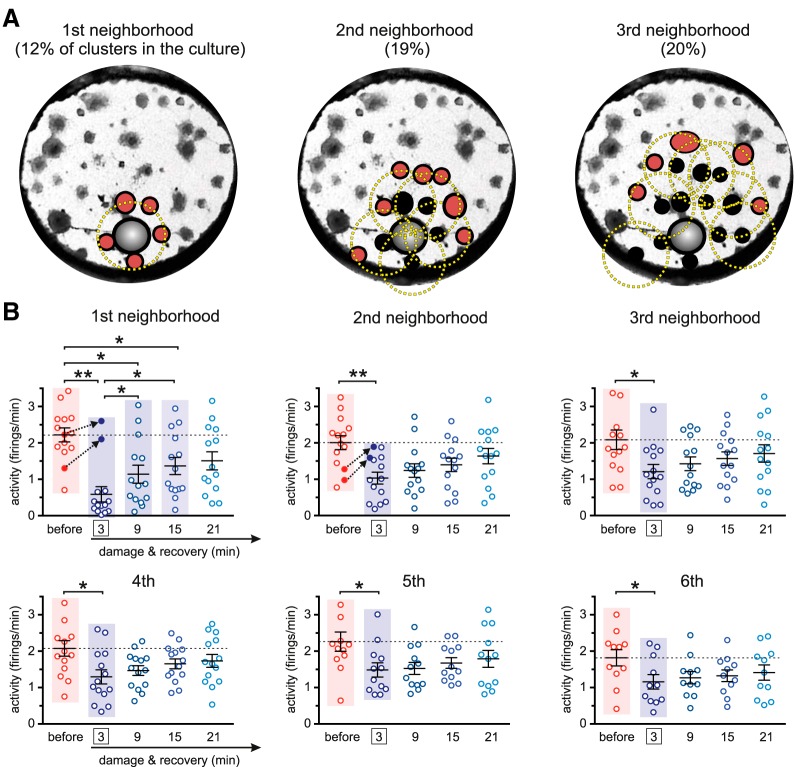
Spontaneous activity in the neighborhood of damage. ***A***, Construction of the ring of neighbors for a representative culture. The first neighborhood ring (red clusters) is constituted by all clusters whose centers fall within a distance $R_{C}=0.68$ mm (yellow circle) from the ablated cluster (gray). The second and subsequent rings are built by identifying the clusters that are neighbors of the clusters in the previous ring according to the same distance *R_C_*. ***B***, Box plots showing the temporal evolution of the average activity in six neighborhoods and for the 14 experimental realizations. Before damage, activity is averaged over 30 min. The indicated times correspond to the center of the analysis windows *t*. For damage (at *t* = 3 min, boxed) and subsequent recovery stages, activity is averaged in 6-min windows. Dotted black arrows highlight two experiments whose activity boosted up after damage. Average values of the distributions are shown as mean ± SD. The colored panels highlight the distributions that are significantly different according to a one-way ANOVA (*p < 0.05, **p < 0.01).

The results shown in Figure 3*A* evinced the pivotal role of clusters’ activity during recovery, which translated into the emergence of strong effective links at the vicinity of damage. To better understand this role, we investigated clusters’ activity in progressively distant neighborhoods with respect to the damaged region. As illustrated in Figure 4*A*, the clusters that shaped the ring of “first neighbors” were those centered at a distance below a characteristic radius $r_{C}≃0.68$ mm from the ablated cluster. The second and further neighborhood rings were formed by those clusters located at a distance *r_C_* from the previous ring and away from damage.

For each neighborhood we plotted the average clusters’ activity at different time steps, and encompassing all 14 experimental realizations. As shown in Figure 4*B*, we inspected activity before damage, just after damage, and along different recovery stages. The analysis of the data procured two major results. On the one hand, damage had a strong impact on activity in all neighborhoods, as indicated by the significantly different distributions of clusters’ activity values before and after damage (*p* < 0.05, one-way ANOVA). Activity dropped by 74%, 49%, and 41% for the first, second, and third neighborhoods, respectively. The remaining neighborhoods reduced activity by 30%. On the other, once recovery took action, activity in the neighborhoods gradually increased along time, although at different rates. The first and second neighborhoods, for instance, boosted activity by 100% (from 0.6 to 1.2 firings/min) and 30% (from 1.0 to 1.3 firings/min), respectively, 9 min after damage. The other neighborhoods also increased activity on average, but by a milder 10–20%.

Activity data also showed experiment-specific traits that are worth pinpointing. Specifically, we identified two experiments in which activity in the first and second neighborhoods increased after damage (Fig. 4*B*, black arrows), and that suggests a sudden rerouting of activity flow across the network. This result illustrates the complexity of physical damage in neuronal circuits and the intricate structure–function relationship, in which local direct loss of neurons or synaptic connections does not necessarily trigger a cascade of failure at the vicinity of damage.

### Interaction among neighborhoods during recovery

**Figure 5.**
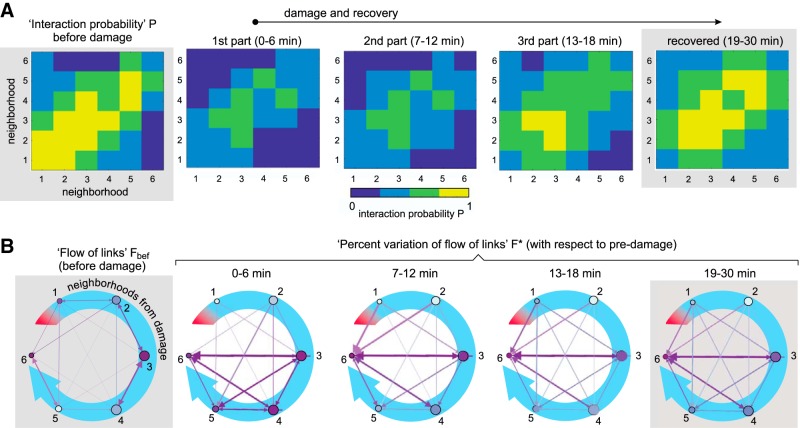
Network communication during recovery. ***A***, Interaction probability *P* among all pairs of neighborhoods. The brighter the color, the higher the formation of effective links between and within neighborhoods. Data were computed for each culture and then averaged over the 14 studied cultures. The left panel shows the neighbors’ interaction before damage, with data averaged over 30 min. The three central panels show the action of damage and subsequent recovery, with each panel corresponding to ∼6-min window intervals for analysis. The last panel shows the stationary recovery, with data averaged over a broader window of ∼12 min. ***B***, Corresponding representation of the flow of links $F_{\text{bef}}$ (before damage) and the percentage variation of flow of links F^*^ (rest of panels, calculated with respect to the predamage scenario). The blue curved arrow and the numbers indicate the distance from damage in terms of neighborhoods. Damage locus is symbolized as a red band. Before damage, purple arrows depict the communication flow between neighborhoods. After damage, the arrows depict the level of formation of new effective links between neighborhoods with respect to predamage. Arrows’ thickness and color intensity are coded according to the values of *F* or F^*^. Nodes’ color is coded according to the relative strength (weighted sum of incoming and outgoing links) of the neighborhood.

To complete the picture and gain further insight on the recovery process, we studied the degree of interaction within and between neighborhoods. This interaction was quantified through the probability *P* of observing new effective connections among clusters that belong to the same or different neighborhoods. Data were computed for each culture and then averaged over cultures. As shown in Figure 5*A*, clusters’ interactions before damage were strong and localized. Each ring of clusters exhibited a dense internal effective connectivity and was strongly coupled with its immediate neighbors. Damage caused an overall fall of clusters’ interactions that affected most prominently the first and second rings around the ablated cluster. The third and fourth rings maintained a high degree of internal and external interactions, which gradually extended toward the rest of the network as recovery took place. These results suggest that the intermediate regions of the network, i.e., those that are neither too close nor too far from damage, initiated the recovery process. Remarkably, once recovery was attained, the interaction within and between neighborhoods was very similar to the one before damage. Only the first ring deviated from this trend. We argue that this ring was substantially sensitive to the physical wiring and activity drive of the ablated cluster, therefore substantially hampering ring’s recovery.

Given the importance of interneighborhood communication during recovery, we analyzed in more detail the flow of effective links between neighborhoods. Figure 5*B* portrays the directionality and degree of formation of effective links among neighborhoods. Before damage, the flow of links $F_{\text{bef}}$ shows that all six rings interacted among themselves with a similar degree, a result that is in agreement with the high coordinated activity of the clusters in the network. The action of damage, emphasized here by plotting the variation of flow of links F^*^ with respect to the predamage condition, broke the uniformity of communication between neighborhoods and made ring 3 the leader in the formation of new effective links. Indeed, as the panels of Figure 5*B* show, the beginning of recovery was characterized by a substantial flow of new links that either diverged from ring 3 or converged toward it, and essentially involving rings 4–6. This behavior reinforces the message that areas neither too close nor too far from damage (as ring 3 in our case) play a substantial role in maintaining network activity and leading recovery. In our experiments, as recovery progressed, rings 1 and 2 gradually participated more actively, shaping new effective links toward the rest of the network and balancing the entire system again. At full recovery, the interaction between neighborhoods procured an almost uniform formation of new links.

## Discussion

The *in vitro* model presented here takes advantage of the accessibility and ease of manipulation of clustered neuronal cultures to investigate network’s recovery and functional reorganization after acute focal damage. Our results show that the neuronal clusters adjacent to the lesion core, first and second neighborhood rings, were the most affected by damage, possibly due to the loss of direct physical connections. However, damage did not precipitate a cascade of failure. Clusters weakly linked to the lesion core, third and fourth rings, coped with damage and led recovery by establishing new functional connections or strengthening existing ones. Dynamic interactions extended next to the whole network to restore its communication to predamage levels.

In the experiments, the ablated cluster was chosen arbitrarily, and with the only condition that its level of activity was similar to the average of the network. For the 14 cultures studied, in 60% of the experiments the ablated cluster was located at the edge of the culture, and for the remaining 40% it was located at the center. We could not pinpoint any significant correlation between the location of the ablated cluster and the characteristics of recovery, indicating that the distance of the clusters to the damage core was the main factor shaping the initial functional loss and subsequent restoration. On average, the damage locus comprised a circular area 100 μm in diameter that accounted for ∼3% of all neurons in the network, which were irreversibly lost. The directly affected regions were those located ∼0.7 mm from damage. Recovery was initiated in regions ∼1.5 mm away, and functional restoration reached the affected regions in ∼15 min.

Reorganization of brain circuits after damage involves structural and functional changes that compensate for both the lesion itself and remote effects in the brain (van Meer et al., 2012; Jiang et al., 2013; Grefkes and Fink, 2014; Siegel et al., 2016). In our experiments, however, it is unlikely that structural remodeling of intercluster connectivity is the major mechanism underlying the observed network recovery given the short time scales involved. Although axonal growth and formation of new synaptic connections are fast processes (Marrs et al., 2001; Malyshevskaya et al., 2013), few hours would be required in our preparations to bypass the lost neuronal cluster and rewire the neighboring clusters among themselves, which are typically 1 mm apart. This time scale of hours is far beyond the 15 min observed for recovery. Thus, we hypothesize that the central role of the physical network after damage is to support the rerouting of intercluster dynamic interactions, reshaping information flow and functional reorganization. On recovery, the major traits of the functional network, namely global efficiency and density of effective links, were similar to the predamage condition. This suggests that the neuronal network not only restored function, but that regulated itself to secure adequate operation levels. Such homeostatic mechanisms have been reported in studies of brains affected by lesions (Butz-Ostendorf and van Ooyen, 2017), and have been hypothesized to play a central role at early stages of recovery.

Despite the differences between *in vitro* and *in vivo* systems, our results are in striking accordance with previous studies in mouse brains. Lim and collaborators (Lim et al., 2014) followed network activity with a voltage sensitive dye on optogenetic stimulation of different areas after cortical damage by stroke. They showed that the extent of network affection depended on the connectivity strength between the monitored brain areas and damage locus. Nearby, peri-infarct areas were severely affected, whereas more distant, weakly connected areas remained unaltered. Lim’s study also showed that recovery initiated in distant areas and progressed heterogeneously toward the infarcted region. Despite the different size and time scales of Lim’s work as compared to ours, the similarity between the studies illustrates the importance of connectivity-based approaches to investigate recovery after local acute damage (Zhu et al., 2010; Grefkes and Fink, 2014). *In vitro* experiments in combination with network analyses thus provide an excellent platform to relate connectivity failure with functional alterations and recovery mechanisms. The gap between *in vivo* and *in vitro* architectures can be reduced through neuroengineering, which allows to mimic major organizational (Aebersold et al., 2016) and dynamical (Yamamoto et al., 2018) features of brain circuits while maintaining full access to neurons and connections. The analysis of damage and recovery in these advanced designs will open new avenues for understanding the link between complex network topologies, damage and functional resilience, more prominently in the context of modular organization (Sporns and Betzel, 2016), and node centrality (Alstott et al., 2009; Fornito et al., 2015).

The mechanisms of recovery observed in our study have important clinical implications. Cheng and coworkers (Cheng et al., 2014) demonstrated that optogenetic stimulation of cortical areas located in the vicinity of a stroke-injured mouse brain promoted overall activity and enhanced multiple plasticity-associated mechanisms, which altogether fostered whole-brain functional restoration. Translated to our *in vitro* model, such stimulation protocol would correspond to induce activity in the first and second neighborhood rings around the ablated cluster, which would possibly accelerate functional recovery. Our *in vitro* system is highly tunable and stimulation protocols of different nature, optogenetic, electrical, or chemical, can be easily integrated. In combination with our present capacity of precise surgery and whole-network monitoring, stimulation approaches could facilitate a deeper comprehension of the processes underlying network reorganization during recovery, and could foster the development of new therapeutic strategies in affected brains.

To conclude, we emphasize that the restitution of damaged circuitry and overall functional remodeling constitute central mechanisms to prevent a fatal cascade of failures or the complete inoperability of neuronal networks. Our work shows that functional remodeling is fast and robust. Following the recent study of Harush and Barzel (2017), we advocate that dynamic reorganization and the access to diverse pathways for information flow are much more important for resilience than previously thought. Our *in vitro* approach brings new experimental opportunities and opens new frontiers to comprehend the intricacy of dynamic interactions and functional reorganization in complex networks.

## Synthesis

Reviewing Editor: Gustavo Deco, Universitat Pompeu Fabra Decisions are customarily a result of the Reviewing Editor and the peer reviewers coming together and discussing their recommendations until a consensus is reached. When revisions are invited, a fact-based synthesis statement explaining their decision and outlining what is needed to prepare a revision will be listed below. The following reviewer(s) agreed to reveal their identity: Gianluigi Mongillo.

Reviwerer 1:

The authors develop a novel in vitro experimental setting in which the re-organization of patterns of neuronal activity following localized damage to cultured networks can be carefully investigated. The main experimental observation is that, in clustered neuronal cultures, following the ablation of one of the clusters, the statistics of neuronal activity spontaneously recover over a time scale of about 15 minutes. The authors then characterize in quite some detail, the spatial and temporal evolution of this spontaneous recover. As the authors state in the manuscript, this is indeed an interesting setup in which theories about spontaneous recovery (following damage) in neuronal networks can begin to be tested.

The results presented are novel and no doubt interesting and, as I have already said, the experimental setup appears quite promising. The manuscript is well written. I have, however, a few concerns mostly about the data analysis.

I was a little bit confused as to whether the analyses are carried out with the effective connectivity (defined in Eq. (1)) or with the raw connectivity (i.e., the w_ij). The reason is that effective connectivity is denoted z_ij in Eq. (1), but then all other observables are defined with the w_ij (e.g., the nodal strength s_i). Please clarify this point.

I am not sure I understand the logic behind Eq. (1). Naively, I would think that the point of having surrogate matrices is to assess significance of the w_ij, with the purpose of removing the non-significant links for further analysis (e.g., setting to 0 all links that are within 3 SDs from the surrogate averages). However, this is not what is achieved by Eq. (1), which simply rescales the weights (which then are rescaled once more (pag. 7, lines 149-150). Moreover, an artificial asymmetry is introduced when negative z_ij are set to zero (pag. 7, lines 148-149). It is unclear what is the impact of this 'rectified' rescaling of the w_ij's on further analysis. Please clarify this important point.

Along the same lines, I have found the way control-corrected statistics are introduced and used not fully satisfactory, mostly because the pairing between control and ablated is somehow “arbitrary”. Note that it could be that each control differs from its corresponding ablated, and yet these differences could disappear when averaged over the population (see, e.g., Fig 3A, before damage). It would seem, however, that this pairing is not really necessary. One could just compute “null” statistics on the control networks and use these to assess significance of the corresponding statistics in the ablated networks, without pairing. Please address this point.

Directly following from the above point, it would be interesting to know how stable (in time) are the different statistics in the control population, such as, global efficiency, density of links and activity level of given clusters.

Reviewer 2:

This manuscript demonstrates that the progress of functional recovery of the neural circuit at the network levels after focal ablation by laser micro-surgery. Their Methods for evaluation of network's and culturing of neurons would be useful for multiple fields that engineering, pharmacology, etc. Although this is a well-written paper and is suitable for publication in eNeuro, I have few comments to improve the manuscript.

In Figure 4B the author used a t-test, but it should be used ANOVA to avoid multiple comparison problems.

In Figure 5 B, I think the link between neighborhoods 3 and neighborhoods 6 was predominant, but in the figure 5A there is very low probability between 3 and 6. I would like to know the reason for this discrepancy.

Line 90commercial confocal microscope (NIkon C1) Typo? Nikon -> Nikon
